# Consistent cord blood DNA methylation signatures of gestational age between South Asian and white European cohorts

**DOI:** 10.1186/s13148-024-01684-0

**Published:** 2024-06-06

**Authors:** Wei Q. Deng, Marie Pigeyre, Sandi M. Azab, Samantha L. Wilson, Natalie Campbell, Nathan Cawte, Katherine M. Morrison, Stephanie A. Atkinson, Padmaja Subbarao, Stuart E. Turvey, Theo J. Moraes, Piush Mandhane, Meghan B. Azad, Elinor Simons, Guillaume Pare, Sonia S. Anand

**Affiliations:** 1 Peter Boris Centre for Addictions Research, St. Joseph’s Healthcare Hamilton, Hamilton, Canada; 2 Department of Psychiatry and Behavioural Neurosciences, McMaster University, Hamilton, Canada; 3 Department of Medicine, Faculty of Health Sciences, McMaster University, Hamilton, Canada; 4 Population Health Research Institute, David Braley Cardiac, Vascular and Stroke Research Institute, Hamilton, Canada; 5 Thrombosis and Atherosclerosis Research Institute, David Braley Cardiac, Vascular and Stroke Research Institute, Hamilton, ON Canada; 6 Department of Health Research Methods, Evidence, and Impact, McMaster University, Hamilton, Canada; 7 Department of Obstetrics and Gynecology, McMaster University, Hamilton, Canada; 8 Department of Pediatrics, McMaster University, Hamilton, Canada; 9 Hospital for Sick Children, Department of Pediatrics, University of Toronto, Toronto, Canada; 10 Program in Translational Medicine, SickKids Research Institute, Toronto, Canada; 11 Department of Pediatrics, BC Children’s Hospital, The University of British Columbia, Vancouver, Canada; 12 Department of Pediatrics, University of Alberta, Edmonton, Canada; 13 Department of Pediatrics and Child Health, Children’s Hospital Research Institute of Manitoba, University of Manitoba, Winnipeg, Canada; 14 Section of Allergy and Immunology, Department of Pediatrics and Child Health, University of Manitoba, Winnipeg, Canada; 15 Department of Pathology and Molecular Medicine, Michael G. DeGroote School of Medicine, McMaster University, Hamilton, Canada

**Keywords:** Cord blood DNA methylation, Gestational age at birth, Epigenetic gestational age, Accelerated gestational age

## Abstract

**Background:**

Epigenetic modifications, particularly DNA methylation (DNAm) in cord blood, are an important biological marker of how external exposures during gestation can influence the *in-utero* environment and subsequent offspring development. Despite the recognized importance of DNAm during gestation, comparative studies to determine the consistency of these epigenetic signals across different ethnic groups are largely absent. To address this gap, we first performed epigenome-wide association studies (EWAS) of gestational age (GA) using newborn cord blood DNAm comparatively in a white European (*n* = 342) and a South Asian (*n* = 490) birth cohort living in Canada. Then, we capitalized on established cord blood epigenetic GA clocks to examine the associations between maternal exposures, offspring characteristics and epigenetic GA, as well as GA acceleration, defined as the residual difference between epigenetic and chronological GA at birth.

**Results:**

Individual EWASs confirmed 1,211 and 1,543 differentially methylated CpGs previously reported to be associated with GA, in white European and South Asian cohorts, respectively, with a similar distribution of effects. We confirmed that Bohlin’s cord blood GA clock was robustly correlated with GA in white Europeans (*r* = 0.71; *p* = 6.0 × 10^–54^) and South Asians (*r* = 0.66; *p* = 6.9 × 10^–64^). In both cohorts, Bohlin’s clock was positively associated with newborn weight and length and negatively associated with parity, newborn female sex, and gestational diabetes. Exclusive to South Asians, the GA clock was positively associated with the newborn ponderal index, while pre-pregnancy weight and gestational weight gain were strongly predictive of increased epigenetic GA in white Europeans. Important predictors of GA acceleration included gestational diabetes mellitus, newborn sex, and parity in both cohorts.

**Conclusions:**

These results demonstrate the consistent DNAm signatures of GA and the utility of Bohlin’s GA clock across the two populations. Although the overall pattern of DNAm is similar, its connections with the mother's environment and the baby's anthropometrics can differ between the two groups. Further research is needed to understand these unique relationships.

**Supplementary Information:**

The online version contains supplementary material available at 10.1186/s13148-024-01684-0.

## Background

Epigenome-wide DNA methylation (DNAm) patterns across a variety of tissues and cells have been shown to accurately capture the “biological clock” in both adult [1–3] and pediatric populations [4–6], providing insights into an individual's biological age, which can differ from their chronological age. These epigenetic clocks and their deviations from chronological age, otherwise known as accelerated aging, have been linked to a range of health outcomes and age-related diseases, suggesting that DNAm changes serve not just as markers of age, but also play a role in the mechanisms of aging that overlap with many chronic diseases [7, 8].

Chronological gestational age (GA) at birth—the actual time elapsed since the last menstrual period of a pregnant woman—is a fundamental component of neonatal care. Babies born preterm (before 37 weeks) or post-term (after 42 weeks of gestation) may have increased risks for various health conditions later in life [9–11], such as cardiovascular diseases, respiratory issues, and metabolic syndrome. In turn, maternal exposures, such as the mother’s health and lifestyle choices, many of which are modifiable, can also influence GA [12, 13]. On the other hand, biological GA at birth has been conceptualized as a measurement of fetal development that can deviate from the chronological gestational age. It is typically measured using biomarkers, such as epigenetic [5] or metabolic markers [14], which allow for a more nuanced understanding of fetal development and the impact of maternal exposures. In particular, epigenetic GA, which can be determined by DNAm GA clocks, has emerged as a more precise approach to measuring biological maturation in response to *in-utero* environmental exposures with potential predictive value for children’s future development and health.

Considering that DNAm patterns are tissue-specific and can reflect gene expression changes, maternal exposures can act as potential triggers to induce tissue-dependent DNAm alterations. For example, nutrition, cigarette smoking, alcohol use, parity, education, and physical health during gestation have been linked to DNAm shifts measured in the placenta [15–18] and newborn cord blood [19–37]. These exposures can reshape the epigenetic landscape of the fetus and placenta, and influence health in later life [38–42]. Further, a pronounced acceleration in GA (GAA), measured by how much the predicted epigenetic age is higher than the chronological age at birth, has also been associated with maternal exposures and offspring characteristics, including older maternal age, a higher pre-pregnancy body mass index (BMI), maternal smoking, gestational diabetes mellitus (GDM), pre-eclampsia, mode of delivery, higher birth weight and length, as well as male sex in newborns [28, 43–50]. Meanwhile, placental and cord blood DNAm can provide complementary but distinct information, as placental DNAm reflects the interface between mother and fetus and its unique adaptations, while cord blood DNAm is more representative of the systemic fetal epigenetic profile at birth. Consequently, epigenetic clocks based on placental DNAm data do correlate with those derived for cord blood, but differ in terms of the CpGs included, with very little overlap [18], possibly due to the transient nature of placenta tissue and how specific tissues respond to environmental exposures.

The majority of studies on epigenetic GA, GAA, and the characterization of their associations focused on white Europeans. In particular, epigenetic GA clocks were derived almost exclusively in white European populations [5, 6, 51–53] and knowledge about their performance in non-white populations is limited. The general lack of ethnic and racial diversity in omics research restricts the generalizability of these findings. Certain racial groups, such as Black and Asian, are at a disadvantage as they have on average a shorter gestation duration [54] and are at a higher risk for pregnancy complications [55, 56]. South Asian women, owing to their distinct genetic backgrounds [57], environmental exposures [58], and socio-cultural practices [59], are nearly two times more likely to develop GDM as compared to white European women [60, 61]. These genetic and environmental differences become especially pronounced in the length of gestation and its relationship with birth weight. Non-white populations, with their distinct set of genetic and environmental exposures, may present a different profile of epigenetic GA and GAA, which warrants a comparative study of the associated characteristics among diverse populations.

Here, we propose to comparatively examine EWAS of GA and association studies of epigenetic GA and GAA between white European and South Asian birth cohorts with all participants living in Canada. First, to understand whether differences exist between the two populations, we contrasted characteristics of DNAm signatures at individual CpG levels using EWASs, and then at an aggregated level using epigenetic GA and GAA between the two populations. Second, to gain insights on whether these epigenetic markers are differentially or similarly influenced by prenatal factors in the two populations, we comprehensively examined associations of epigenetic GA, and GAA with an extensive collection of maternal exposures and offspring outcomes.

## Methods

### Study designs

CHILD is a prospective longitudinal birth cohort [62] that enrolled > 3,600 pregnant women who gave birth between 2009 and 2012, in Vancouver, Edmonton, Winnipeg, and Toronto, Canada. The SouTh Asian biRth cohorT (START) study is a prospective longitudinal birth cohort [63] that focused exclusively on people who originated from the Indian subcontinents known as South Asians. START recruited South Asian women between 18–40 years of age who were pregnant with a single fetus from the Peel region in Ontario, Canada between 2011 and 2013. These cohorts were not enriched for any clinical conditions and only singleton mothers were recruited. Our analyses focused on a subset of white European-origin mother–offspring pairs from CHILD and South Asian mother–offspring pairs from START, all of whom provided cord blood samples for genome-wide DNA methylation profiling.

### Cord blood DNA methylation data in CHILD and START

Data quality processing has been described previously [64]. Briefly, 997 cord blood samples were hybridized to the Illumina Human-Methylation450K BeadChip (HM450K) array, covering CpG or 5'—C—phosphate—G—3' sites in the entire genome [65]. We followed standard quality control procedures designed for HM450K using the R “*sesame*” package [66] and generated the β-matrix for further processing. Suppl. Table 1 summarizes the sample and probe inclusion/exclusion criteria. Briefly, duplicated probes, probes with detection threshold *p*-value < 0.05, more than 10% missing, or known to be cross-reactive or overlap with single nucleotide polymorphisms were removed [67, 68]. Further, for CpG probes with a missing rate < 10%, mean imputation was used to fill in the missing values. At each CpG site, the *β*-value reflects the ratio of methylated signals relative to total signals and is a continuous measure between 0 and 1. The cleaned datasets contain 504 START samples with 361,234 CpGs and 352 CHILD samples with 358,113 CpGs, covering CpG sites on autosomes and X chromosomes. Finally, we estimated cell-type proportions (CD8T, CD4T, Natural Killer cells, B cells, monocytes, granulocytes, and nucleated red blood cells) following a reference-based approach developed for cord blood [69] using the R package “*FlowSorted.CordBloodCombined.450 k*”.

### Maternal variables

Participants in the START and CHILD cohorts completed self-reported, study-specific questionnaires, which were used to derive standardized variables on personal and family medical histories, as well as social and cultural practices. Maternal diet was assessed using a previously validated ethnic-specific food frequency questionnaire (FFQ; [70] in START and a semi-quantitative multiethnic FFQ (created by the US-based Fred Hutchinson Cancer Center) in CHILD [70].

Three validated maternal diet patterns [71] were derived from the FFQs: 1) the plant-based pattern was characterized by vegetables, legumes, fermented dairy, whole grains, nonmeat dishes, and a lack of red meat; 2) the Western pattern had a high loading of sweets, red and processed meats, French fries, starchy vegetables, condiments, and sweet drinks; and 3) the health-conscious pattern was characterized by seafood, poultry, and red meats; eggs; cruciferous vegetables; leafy greens; fruit; refined grains; stir-fried dishes; and condiments. Maternal smoking history (current, quit during pregnancy, quit before pregnancy, never-smoker) and household smoking exposure (hours per week) were self-reported. Maternal education questions were harmonized between CHILD and START to produce a single variable indicating the number of years in school. Socioeconomic status was captured by the validated social disadvantage index, a composite measure of household income, marital status, and maternal employment [72], and was weakly correlated with maternal education (*r*^*2*^ < 0.05). Clinical variables of interest included maternal age at delivery, pre-pregnancy BMI (kg/m^2^), pre-pregnancy weight (kg), gestational weight gain (kg), GDM status, gestational hypertension, pre-eclampsia, and parity (defined as the number of births before current pregnancy). GDM was determined by self-report only in CHILD, while in START, it was determined by a combination of oral glucose tolerance test (OGTT), self-report, and reported diabetic treatments, including insulin, pills, and restricted diet. The OGTT threshold (> = 5.2 at baseline or >  = 7.2 at 2 h) for South Asian women proposed in Born in Bradford [73, 74] was used in START.

### Offspring characteristics

Chronological GA at birth, reported in weeks and days, was collected from participants’ birth charts. As an additional quality control step, we excluded 10 newborns from CHILD and 16 from START who either lacked GA data or were born before 36 weeks, such that the remaining newborns had gestation at birth between 36 and 42 weeks. Offspring anthropometrics are available at birth from medical charts or measured at < 2 days old, and subsequently measured at the 1, 2, 3, and 5-year follow-up visits. These included height (cm), weight (g), ponderal index (PI; kg/m^3^), and BMI (kg/m^2^), at all visits; and the sum of skinfolds (triceps and subscapular skinfolds in mm), waist circumference (cm), hip circumference (cm), and waist-hip ratio (WHR) at 3- and 5-year visits.

### EWAS of GA

The final datasets with cleaned DNAm and chronological GA at birth consisted of 342 and 490 mother-newborn pairs from CHILD and START, respectively. We performed an EWAS separately in each cohort, testing the association between DNAm *β*-values at each CpG and GA using a linear regression model. Contrary to existing EWASs where the methylation values are typically treated as the outcome and the exposure as the predictor; we reversed the regression such that the methylation levels were the predictors and chronological GA at birth as the outcome. This reverse regression approach is statistically more robust as GA is roughly normally distributed and produces similar evidence of association between the two sets of variables as the original approach [75]. Further, this reverse regression is consistent with our subsequent analysis using DNAm GA clock, a linear combination of additive effects over multiple CpGs, as the predictor and chronological GA at birth as the outcome. Though neither the plate or the row number, the two main sources of batch effects [76], was associated with GA using an omnibus test, there was more variability in the pairwise differences of GA between plate numbers (*p* = 0.005–0.94 in CHILD and *p* = 0.01–0.94 in START). Among the subset of samples with genetic data, there was no association between the first 10 genetic principal components and chronological GA at birth in START (adjusted *R*^2^ = − 0.0057, *n* = 480, *p*-value = 0.7) or CHILD (adjusted R^2^ = 0.0022, *n* = 290, *p*-value = 0.4). Considering the reduced sample size and the absence of evidence indicating confounding, we concluded that further adjustment for genetic ancestry within each cohort was not necessary. Thus, the final model adjusted for the plate number to correct for potential batch effects, the estimated compositions of cord blood cell types, maternal age, maternal education, maternal smoking history, social disadvantage index, parity, and newborn sex (coded as 0 for male and 1 for female). For association testing of X chromosome CpGs, we adopted a robust 2 degrees of freedom test proposed for X chromosome genetic associations to mitigate the influence of unknown X-inactivation [77]. We then meta-analyzed association results from CHILD and START using an inverse variance-weighted fixed-effect model, and CpGs that showed heterogeneity in effects were retained for further clarification. CpG sites located within genes have been labeled using the UCSC reference gene names provided by the annotation file from Illumina (‘HumanMethylation450_15017482_v.1.2.bpm’). For each EWAS or meta-analysis, the false discovery rate (FDR) adjustment was used to control for multiple testing and an FDR-adjusted *p*-value < 0.05 was considered statistically significant. We further contrasted our primary findings with CpGs that were previously linked to GA using cord blood DNAm as reported in Bohlin [51]. The concordance between signals from Bohlin and each EWAS, and those between START and CHILD, was tested using a two-sample proportion test.

### EWAS functional analysis

Differentially methylated regions (DMRs) were identified using DMRcate [78], which is a data-driven method that incorporates only spatial annotations. The algorithm combines robust estimates at individual CpG sites via a Gaussian kernel within a given window to produce a smoothed estimate. We followed the recommended default parameters (e.g., ≥ 2 CpGs, Gaussian kernel bandwidth of 1000) to call DMRs. A DMR was deemed significant if the observed smoothed estimate was more extreme than its expected value given the same spatial information at an FDR-adjusted Stouffer's *p* < 0.05. Using genes that overlap with significant DMRs called in CHILD, START, and additionally, genes mapped from significant CpGs in Bohlin et al., we examined for enrichment in biological pathways using g:Profiler [79] (https://biit.cs.ut.ee/gprofiler/snpense; accessed on March 28, 2024) based on several databases, including gene ontology (GO), Kyoto encyclopedia of genes and genomes (KEGG), Reactome, and WikiPathways. HGNC gene symbols that had multiple ensemble IDs match were removed from the query. We deemed a pathway to be relevant if the adjusted *p*-value < 0.05. Finally, special consideration was given to the intersection of genes associated with type 2 diabetes (T2D) and GDM, informed by our preliminary findings on the EWAS and epigenetic GA clock. Specifically, we investigated whether significant CpGs were enriched in T2D and GDM genes, by searching the GWAS catalog [80] for genetic variants associated with T2D or GDM at genome-wide significance (*p* < 5 × 10^–8^). We only selected genetic variants reported in studies with a discovery sample size > 100,000 and published in the last five years, using the keywords “type 2 diabetes mellitus” (EFO ID: MONDO_0005148) and “gestational diabetes” (EFO ID: EFO_0004593). This approach not only ensures that our search reflects the most recent scientific findings but also assumes that any previously established relevant genetic variants would be sufficiently represented and validated within these large, well-powered studies.

### Epigenetic GA and GAA

Epigenetic GA was estimated using Bohlin’s cord blood [51], Knight’s cord blood [5], and Mayne’s placenta [50] GA clocks developed for HM450K data, as well as Lee’s placenta [6] and Haftorn’s cord blood [52] clock for EPIC data. These algorithms are implemented in the R package “methylclock” [81]. GAA was calculated as the residual from a linear regression using the estimated epigenetic GA as the outcome and the chronological GA as the predictor such that GAA would be uncorrelated with epigenetic GA. A positive GAA indicates acceleration whereby the newborn’s epigenetic GA is older than the chronological GA, and a negative value implies deceleration. We empirically assessed whether the GA clocks and the corresponding GAA were transferrable to the START cohort by examining the distribution of these scores between START and CHILD using a* t*-test.

### Statistical analysis

The performance of various GA clocks to predict chronological GA was quantified using Pearson’s correlation coefficient in both cohorts. The GA clock that most strongly correlates with the chronological GA was used as the estimated epigenetic GA in all subsequent analyses. We then explored maternal characteristics that were predictive of GA, epigenetic GA, and GAA using a stepwise model selection method. The search would yield a final deterministic model with a subset of the variables as evaluated by the Akaike information criterion. For completeness, we further tested the univariate association between GA, epigenetic GA, and GAA and individual traits in the following categories: 1) newborn sex, and birth anthropometrics [4 variables]; 2) offspring anthropometrics at each follow-up visit [20 variables]; and 3) maternal exposures [14 variables], using simple linear regression. An FDR adjustment was applied to univariate results and *q*-values were reported whereby we claimed suggestive evidence of association when *q* < 0.1. For offspring anthropometrics, whenever appropriate, the model adjusted for children’s age and sex at each visit. The reported effect sizes were based on the original scale of each variable for ease of interpretation. Missing data were imputed using a random forest imputation algorithm implemented in the R package “missforest” [82]. All data processing and analyses were conducted in R v.4.1.0 [83].

## Results

### Study sample characteristics

Demographic characteristics and relevant covariates of the epigenetic subsamples (Table 1) were not statistically different (*p* > 0.001) from their respective full cohort after controlling for multiple hypothesis (Suppl. Table 2). Maternal characteristics, including parity, maternal age, pre-pregnancy weight, GDM, smoking history, education, social disadvantage index, and diet patterns, were significantly different between South Asian and white European women. Specifically, there was a much higher rate of GDM (36% in START vs. 4% in CHILD), and pregnant South Asian women in START were exclusively non-smoking during pregnancy. There was also a noticeable difference in the pattern of phenotypic correlations between the two cohorts, both in size of correlation and directions (Suppl. Figure 1). In terms of newborn characteristics, the mean GA at birth in START (39.3 ± 1.1) was lower than in CHILD (39.6 ± 1.3) when restricted to gestation superior or equal to 36 weeks. As expected, South Asians had a lower birth weight (3.3 ± 0.45 kg vs. 3.5 ± 0.48 kg), but were of similar length as compared to white Europeans. Even after accounting for chronological GA at birth, newborn sex, social disadvantage index, and GDM, the difference in birth weight persisted (*p* < 0.001).

**Table 1:** Sample characteristics of CHILD and START cohorts

	CHILD	START	*P*-value
	(*N* = 342)	(*N* = 490)	
*Maternal*			
Mother's Age			
Mean (SD)	32.7 (± 4.4)	30.1 (± 3.9)	< 0.001
Missing	4 (1.2%)	0 (0%)	
Parity			
Mean (SD)	0.72 (± 0.89)	0.80 (± 0.81)	0.191
Missing	0 (0%)	13 (2.6%)	
Pre-pregnancy BMI			
Mean (SD)	24.7 (± 5.2)	23.7 (± 4.4)	0.0236
Missing	131 (38.3%)	2 (0.4%)	
Pre-pregnancy weight			
Mean (SD)	68.8 (± 15.2)	62.6 (± 11.8)	< 0.001
Missing	126 (36.8%)	0 (0%)	
Gestational weight gain			
Mean (SD)	15.4 (± 6.2)	14.3 (± 7.5)	0.0413
Missing	120 (35.1%)	9 (1.8%)	
Gestational diabetes mellitus			
No	328 (95.9%)	313 (63.9%)	< 0.001
Yes	14 (4.1%)	176 (35.9%)	
Missing	0 (0%)	1 (0.2%)	
Gestational hypertension			
No	326 (95.3%)	479 (97.8%)	0.0801
Yes	16 (4.7%)	11 (2.2%)	
Pre-eclampsia			
No	331 (96.8%)	486 (99.2%)	0.0217
Yes	11 (3.2%)	4 (0.8%)	
Smoking history			
Never smoked	241 (70.5%)	487 (99.4%)	< 0.001
Quit before this pregnancy	68 (19.9%)	1 (0.2%)	
Quit during this pregnancy	17 (5.0%)	1 (0.2%)	
Currently smoking	11 (3.2%)	0 (0%)	
Missing	5 (1.5%)	1 (0.2%)	
Smoking exposure (hr/week)			
Mean (SD)	1.0 (± 7.8)	0.33 (± 2.7)	0.13
Missing	12 (3.5%)	39 (8.0%)	
Years of education			
Mean (SD)	17.0 (± 3.1)	15.8 (± 2.4)	< 0.001
Missing	9 (2.6%)	0 (0%)	
Social disadvantage index			
Mean (SD)	0.41 (± 0.95)	1.8 (± 1.4)	< 0.001
Missing	37 (10.8%)	69 (14.1%)	
Plant-based diet			
Mean (SD)	− 0.48 (± 0.46)	1.6 (± 1.1)	< 0.001
Missing	21 (6.1%)	16 (3.3%)	
Health-conscious diet			
Mean (SD)	0.21 (± 0.81)	− 0.42 (± 0.79)	< 0.001
Missing	21 (6.1%)	16 (3.3%)	
Western diet			
Mean (SD)	− 0.16 (± 0.64)	− 0.51 (± 0.65)	< 0.001
Missing	21 (6.1%)	16 (3.3%)	
*Newborn*			
Gestational age (weeks)			
Mean (SD)	39.6 (± 1.3)	39.3 (± 1.1)	< 0.001
Newborn sex			
Male	187 (54.7%)	234 (47.8%)	0.0581
Female	155 (45.3%)	256 (52.2%)	
Birth length (cm)			
Mean (SD)	51.7 (± 2.5)	51.5 (± 2.6)	0.381
Missing	65 (19.0%)	7 (1.4%)	
Birth weight (kg)			
Mean (SD)	3.5 (± 0.48)	3.3 (± 0.45)	< 0.001
Missing	4 (1.2%)	1 (0.2%)	
Newborn BMI (kg/m2)			
Mean (SD)	13.1 (± 1.4)	12.3 (± 1.4)	< 0.001
Missing	66 (19.3%)	7 (1.4%)	
Newborn ponderal index (kg/m3)			
Mean (SD)	25.4 (± 3.1)	24.0 (± 3.2)	< 0.001
Missing	66 (19.3%)	7 (1.4%)	

### EWAS of GA

**Fig. 1:**
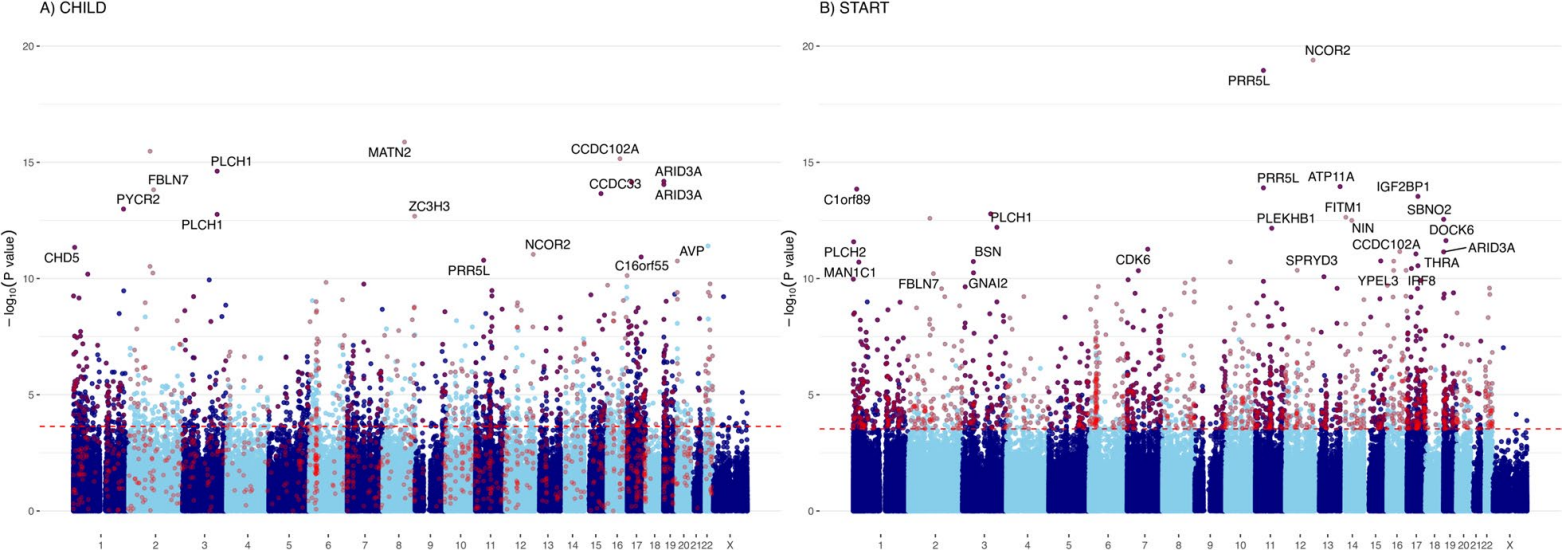
Manhattan plots of the EWAS results of gestational age in CHILD (left) and START (right). Manhattan plots summarized the association *p*-values between cord blood DNA methylation levels and gestational age in CHILD (left) and START (right). The red line denotes the smallest -log10(*p*-value) that is below the FDR correction threshold of 0.05. The red dots represent established associations with gestational age based on ultrasound [51]

Figure 1 highlights the 1,652 and 2,136 differentially methylated CpGs associated with GA after FDR correction (above the red dashed line and in Suppl. Table 3) in CHILD and START, respectively. The number of significant CpGs overlapping between the two cohorts was 599 (Suppl. Figure 2). While the signal overlap between the START and CHILD was smaller than expected, the majority of signals separately from CHILD (73%) and START (72%) agreed with those reported in Bohlin’s EWAS [51] in a much larger sample (*n* > 1000). Across these EWASs, there was considerable empirical inflation at various significance thresholds as well as overall inflation at the medium (Suppl. Table 4), however, the level of inflation was comparable across the studies and had been similarly observed in the context of other -omics studies as a result of widespread true associations [84, 85]. The meta-analysis of all CpGs with heterogeneity* p*-value > 0.01 identified an additional 4664 CpGs (7492 CpGs total) that were significantly associated with GA. Only 108 CpGs that were significant in the meta-analysis showed marginal evidence for heterogeneity of effects (heterogeneity* p*-value < 0.01), however, the estimated effects of these associations were always in the same direction (Suppl. Figure 3). Approximately 78% of the CpGs identified as significant in Bohlin's EWAS (11,337 of the 14,501 reported in the EWAS catalog using ultrasound-estimated GA at birth) were also significant in both CHILD and START. To benchmark our results, we highlighted these CpGs in Fig. 1 in purple. In general, there was good agreement in signals identified between CHILD and START, as well as those between CHILD or START and Bolin’s (Suppl. Figure 4). We identified 1479 and 9142 DMRs in CHILD and START, respectively, following the default FDR adjustment (Suppl. Tables 5 and 6). The largest DMR for CHILD contained 53 CpGs and was mapped to the *B3GALT4* and *WDR46* genes, while the largest DMR for START (5 CpGs) overlapped with the *PRR5L* gene. On average, there was equal representation of significant DMRs across the genome, however, we observed significant DMRs identified in START to contain more CpGs than in CHILD on average (Suppl. Figure 5). At an FDR-adjusted *p*-value threshold of < 0.05, 451 out of 489 pathways identified for CHILD and 675 out of 906 in START were identical to those identified using the significant CpGs from Bohlin (Suppl. Table 7). The top pathways across the three EWASs captured biological processes related to fetal development and immune system function, such as proliferation, migration, differentiation of different types of cells, and ﻿anatomical structure development.

We retained 1509 unique gene regions from the GWAS catalog, of which 1508 were linked to T2D and 7 were associated with GDM. Of these, 130 were represented in genes mapped onto by significant CpGs identified in either CHILD, START, or both (Suppl. Table 8). In both CHILD and START, we found the number of genes containing at least one significant CpG to be overrepresented among known T2D genes (Suppl. Figure 6). Specifically, 71 out of 921 genes identified in CHILD (binomial test for proportion *p* = 9.9 × 10^–5^) and 97 out of 1158 genes identified in START (binomial test for proportion *p* = 1.3 × 10^–7^) were represented in genes reported in T2D or GDM GWASs (Suppl. Table 9).

### Epigenetic GA and GAA

**Fig. 2:**
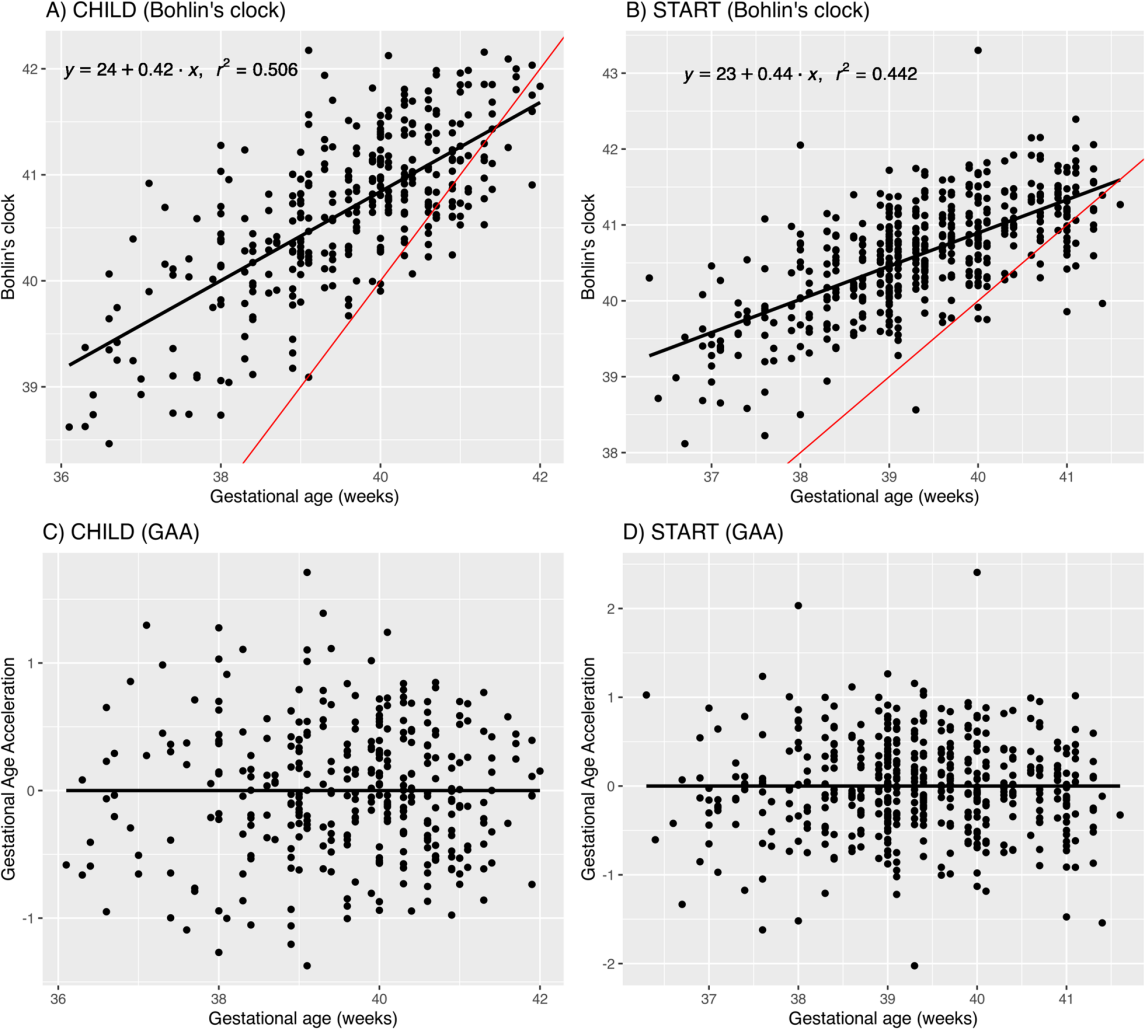
Relationship between newborn epigenetic gestational age at birth based on cord blood DNA methylation data, chronological gestational age at birth, and gestational age acceleration. Panels A-B) show the scatterplot of chronological gestational age at birth and Bohlin’s gestational age clock in CHILD and START, respectively. Panels C-D) show the scatterplot of chronological gestational age at birth and gestational age acceleration in CHILD and START, respectively. The black lines are the best-fitted line using ordinary least squares, with equations displayed in Panels A) and B). The red lines indicate the reference line of y = x

Out of the six epigenetic GA clocks tested, the Knight and Bohlin clocks designed for the HM450 array had better overlap with CpGs in our data as compared to the EPIC clocks with high missingness (Table 2). In terms of performance, the cord blood-based Knight and Bohlin clocks had better correlation with the chronological GA, with the Bohlin clock being consistently the best in START (*r* = 0.66; *p* = 6.9 × 10^–64^) and CHILD (*r* = 0.71; *p* = 6.0 × 10^–54^), producing the highest correlation coefficient and the smallest median absolute difference (Table 2). Thus, all subsequent results were based on epigenetic GA estimated using Bohlin’s cord blood clock. The estimated epigenetic age using Bohlin’s clock and the chronological GA roughly followed a linear relationship, while the residual approach ensured that GAA was uncorrelated with the chronological age (Fig. 2). Finally, there was no statistically significant difference between the mean estimated epigenetic GA in white European (40.6 ± 0.81) and South Asian (40.6 ± 0.74) newborns, respectively. Similarly, there was no difference in the GAA derived using the residual approach (Suppl. Table 10).

**Table 2:** Performance of cord blood DNA methylation gestational age clocks in CHILD and START

Dataset	Clock	Tissue	CpGs used (total)	Pearson's r	*P*-value	MAD (days)
CHILD (n = 342)	Knight	Cord blood	132 (148)	0.60	5.74E-35	9
	Bohlin	Cord blood	79 (96)	0.71	5.98E-54	4
	Mayne	Placenta	54 (62)	0.12	1.18E-02	6
	Lee.RPC	Placenta	950 (1125)	0.20	9.50E-05	6
	Lee.CPC	Placenta	950 (1125)	0.33	1.81E-10	7
	Haftorn-EPIC	Cord blood	71 (176)	0.13	7.73E-03	6
START (n = 490)	Knight	Cord blood	132 (148)	0.44	4.09E-25	14
	Bohlin	Cord blood	79 (96)	0.66	6.90E-64	4
	Mayne	Placenta	54 (62)	0.13	0.0030	11
	Lee.RPC	Placenta	958 (1125)	0.024	0.59	10
	Lee.CPC	Placenta	958 (1125)	0.15	7.40E-04	9
	Haftorn-EPIC	Cord blood	74 (176)	0.028	0.53	8

### Maternal characteristics that are predictive of GA, epigenetic GA, and GAA

The final models for each outcome are summarized in Table 3. Between CHILD and START, the common set of predictors for GA included pre-eclampsia and GDM, but maternal age and dietary patterns were specific to START, while pre-pregnancy weight, BMI, and gestational weight gain were unique predictors for GA in CHILD alone. These models explained 8.03% and 7.90% of the GA variance in CHILD and START, respectively. Meanwhile, parity, GDM, and newborn sex were important predictors in both CHILD and START for epigenetic GA, with gestational weight gain, pre-pregnancy weight and BMI being additionally implicated in CHILD. In particular, GDM was negatively associated with epigenetic GA, but more severely in CHILD with a reduction of 0.66 ± 0.19 weeks as compared to the 0.16 ± 0.07 weeks in START. Together, these predictors explained 8.52% and 4.50% of the epigenetic GA variance, which was higher than that for GA in CHILD but lower than that in START. Both parity and newborn female sex were also negatively associated with GAA in CHILD and START, but GDM was only associated with GAA in CHILD while dietary patterns, gestational weight gain, and maternal smoking were uniquely associated with GAA in START (Table 3).

**Table 3:** Multivariable models using maternal characteristics to predict gestational age, epigenetic gestational age, acceleration of gestational age in CHILD and START

		CHILD								
		GA			DNAm GA			GAA		
		Estimate	Error	*P*-value	Estimate	Error	*P*-value	Estimate	Error	*P*-value
	Intercept	39.73	0.54	2.14E− 209	41.33	0.34	5.10E-281	0.21	0.26	0.43
Predictors	Age (mother)							0.01	0.01	0.068
	Parity	− 0.14	0.07	0.061	-0.14	0.04	0.0015	-0.11	0.03	0.0011
	BMI	− 0.10	0.04	0.017	-0.04	0.02	0.085			
	Weight	0.04	0.01	0.0051	0.015	0.01	0.071			
	Weight gain	0.05	0.01	1.04E04	0.023	0.01	0.0025			
	Diabetes mellitus	− 1.01	0.33	0.0023	-0.66	0.19	7.39E-04	-0.27	0.14	0.055
	Pre-eclamp	− 0.62	0.37	0.096						
	Smoking									
	Smoke									
	Disadvantage									
	Conscious diet									
	Western diet									
	Plant-based diet									
	Newborn sex				-0.15	0.08	0.058	-0.17	0.06	0.0023
Model Metric	*P*-value	6.19E− 06			2.77E− 06			1.71E− 04		
	squared	8.03%			8.52%			5.29%		

		START								
		GA			DNAm GA			GAA		
		Estimate	Error	*P*-value	Estimate	Error	*P*-value	Estimate	Error	*P*-value
	Intercept	41.91	0.64	1.04E− 243	41.90	0.44	3.72E−317	0.25	0.10	0.012
Predictors	Age (mother)	− 0.04	0.01	3.1E04	-0.01	0.01	0.13			
	Parity				-0.11	0.04	0.0095	-0.09	0.03	0.0033
	BMI									
	Weight									
	Weight gain							−0.01	0.00	0.034
	Diabetes mellitus	− 0.34	0.10	8.76E−04	-0.16	0.07	0.019			
	Pre-eclamp	− 0.76	0.53	0.15	-0.58	0.35	0.10			
	Smoking							0.38	0.24	0.11
	Smoke									
	Disadvantage									
	Conscious diet	− 0.14	0.06	0.028						
	Western diet	0.16	0.07	0.034				-0.08	0.04	0.033
	Plant-based diet									
	Newborn sex				-0.11	0.07	0.11	-0.08	0.05	0.084
Model Metric	*P*-value	1.35E− 08			2.51E− 05			0.00056		
	squared	7.90%			4.50%			3.40%		

### Univariate association of maternal and offspring characteristics with epigenetic GA and GAA

Newborns from mothers with GDM on average had a lower epigenetic GA in both CHILD (0.6 weeks, *p* = 2.98 × 10^–3^, *q* = 0.02; Suppl. Table 11) and START (0.18 weeks, *p* = 6.58 × 10^–3^, *q* = 0.039; Suppl. Table 12), but more severely in CHILD as compared to START. Parity was significantly associated with epigenetic GA in both CHILD (*p* = 0.0016, *q* = 0.013) and START (*p* = 1.0 × 10^–4^, *q* = 0.0014), indicating that the number of previous births reduces epigenetic GA. Additionally, in START, the age of the mother was negatively associated with both GA and epigenetic GA (Suppl. Table 12). However, a subsequent subset analysis revealed that maternal age was not associated with epigenetic age when adjusted for parity (*p* = 0.49) and that it was not associated with epigenetic GA when stratified based on the number of previous pregnancies (Suppl. Figure 7). Further, male sex was only associated with GAA in CHILD (*p* = 0.0044; FDR-adjusted *p* = 0.036; Suppl. Table 11) but not in START (*p* = 0.14; Suppl. Table 12).

As expected, the associations between epigenetic GA and newborn weight and length were significant in both South Asian and white European cohorts (*q* < 0.0025; Suppl. Tables 11 and 12). The most interesting contrast was the association with newborn PI, whereby a positive association with GA (0.063 weeks; *q* = 2.89 × 10^–4^) and epigenetic GA (0.034 weeks; *q* = 0.005) was observed in START, but it was negatively associated with GAA in CHILD (0.032 weeks; *p* = 0.0009; *q* = 0.019), despite newborn weight and height being similarly positively associated with GA and epigenetic GA in both cohorts. Both epigenetic GA and GAA were positively associated with 1-year length in CHILD (*q* < 0.02), and additionally GAA with 1-year PI and 2-year length (*q* < 0.05; Suppl. Table 11). We did not observe any association between anthropometrics at subsequent follow-up visits and GA, epigenetic GA, or GAA in START (Suppl. Table 12).

## Discussion

This is the first study to comparatively examine DNAm signatures of GA in two ethnic populations (living in the same country) using cord blood. We found consistent DNAm signatures of gestational age at individual CpG levels and confirmed that Bohlin's epigenetic GA clock correlated well with chronological GA in white Europeans and can be generalized to South Asians. In both populations, the GA clock was positively linked to newborn weight and length, and negatively to gestational diabetes, newborn female sex, and parity. Unique to South Asians, the GA clock was also associated with a higher newborn PI. We confirmed the associations of parity and newborn sex with GAA in both populations and discovered that white European newborn males exhibited twice as much accelerated GA as compared to South Asians.

Previous EWASs of GA have found overlapping signals with other outcomes, such as birth weight, while EWASs of birthweight were enriched for CpGs previously associated with prenatal smoking, folic acid intake, and maternal hypertension or pre-eclampsia [26, 53, 86]. Since the strongest association we found with the epigenetic GA clock was GDM, we were interested in whether GDM or T2D genetics could be implicated in the epigenetics of GA. We found two CpGs significantly associated with GA in START that were present in the *TCF7L2* gene, whose risk alleles increase the risk of T2D by reducing insulin secretion and are also associated with a lower BMI [87, 88]. This lends support to the hypothesis that GDM in South Asians is predominantly due to insulin deficiency, which is consistent with their lower birth weight [89], whereby comparatively white Europeans may have a stronger insulin resistance component. Meanwhile, *GLIS3* is another recognized gene for diabetes that is associated with the development of beta cells [90] and neonatal diabetes [91]. Further, *ADA* gene, which has been linked to T2D [92] and several serum metabolites [93], was also mapped to CpGs significantly associated in START but was just below statistical significance in CHILD. Overall, there was an overrepresentation of genes that were known to associate with T2D in both START and CHILD, but with a more pronounced enrichment in START. The shared genes could be part of the mechanisms through which metabolic risks are transmitted from mothers to offspring, contributing to a transgenerational cycle of metabolic disorders [94–96].

Despite widespread differences in DNAm patterns between South Asians and white Europeans in an adult population [97], at an aggregate level, the Bohlin clock consisting of 132 CpGs produced a robust measure of biological GA with comparable performance in both cohorts. While the epigenetic GA clocks can adequately capture biological GA in both populations, they displayed subtle yet differential associations with maternal exposures and effects on offspring. The multitude of maternal exposures, ranging from nutrition, substance use, and metabolic factors to chronic health conditions, presented a complex tapestry of influences on GA, epigenetic GA and GAA. In general, South Asian women had low to no smoking and a diet that was mostly plant-based, and their newborns often exhibited a lower birthweight and a lower GA as compared to white Europeans. In this particular population, we found evidence of Western dietary pattern to influence GA and GAA. But this should be interpreted in the context of differences in the correlation between two populations: a plant-based diet score was positively correlated with Western and health-conscious diets in South Asian women (*r* = 0.35 and 0.32), but a choice of plant-based diet implied a reduced Western diet (*r* = − 0.21) and a strong preference for health-conscious diet (*r* = 0.64) in white European women (Suppl. Figure 1).

In both cohorts, parity and newborn female sex were found to be negatively associated with epigenetic GA and GAA. While both maternal age and parity have been shown to associate with GAA [18, 49], our univariate and multivariable analyses pointed to parity being independently associated with epigenetic GA and GAA, but not maternal age. Consistent with this claim, a previous study showed a negative association between parity and maternal telomere length, which is an indicator of cellular aging. Advanced maternal age (e.g., > 35) is typically considered a risk factor for pregnancy-related outcomes [98, 99]. Here we showed that maternal age was negatively associated with GA and epigenetic GA in the univariate analysis, but only in the South Asian cohort, suggesting it could be capturing the effect of parity. This was supported by the observation that parity was more strongly correlated with maternal age in START (Pearson’s *r* = 0.45) than CHILD (*r* = 0.27), reflecting a higher proportion of older mothers being multiparous among South Asians. This could also explain why maternal age has the most significant impact on negative outcomes in South Asian women [100]. On the other hand, our finding that newborn males have accelerated GA in both CHILD and START is consistent with established literature that male sex was associated with accelerated aging in newborns [43] and in adult populations [101].

In maternal health outcomes, the observed prevalence of GDM was consistent with those reported for the two populations in general [102, 103]. We observed a negative relationship between GDM and epigenetic GA in both cohorts, as well as GDM and GAA in CHILD. The direction of associations was in line with a previous report [48]. However, there was no evidence to support the role of GDM on GAA in the South Asian cohort despite much higher prevalence and sample size. We were able to replicate previous findings of pre-pregnancy weight and BMI having an impact on epigenetic GA in CHILD [43], but there was a coherent indication that gestational weight gain might be the more relevant exposure to GA and epigenetic GA in both cohorts.

The findings of this study should be interpreted in the light of several limitations. Cord blood DNAm in newborns reflects the regulation of gene activity during embryonic development, cellular differentiation, and response to environmental factors, and is thus a valuable source of information for assessing maternal risk factors and predicting future health outcomes. However, depending on the tissue types, resolution of the epigenetic data (e.g., single cell vs. tissue), technology (HM450K vs. EPIC), and method of GA clock construction, the performance of existing GA clocks varied substantially. The main observation is that the barriers to trans-ethnic EWAS analysis lie in various choices of technical and study design. While tissue-specific clocks generated roughly similar results between the South Asian and the white European cohorts (Table 3), we also observed that the robust Lee clock designed for pregnancies without complications produced very different results, possibly due to the large difference in GDM cases (36% in START vs. 4% in CHILD). For example, different tissue types might reveal tissue-specific responses, a placental clock could produce an elevated association with maternal exposures vs. offspring outcomes [6], which is complementary to existing studies of GA using cord blood. We have shown that DNAm clock, while universal in its foundational concept, can exhibit specific patterns and variations when analyzed across different ethnic groups. Factors contributing to these disparities might range from genetic backgrounds to varied environmental and socio-cultural practices that might not be readily available, such as alcohol consumption [34, 104] and air pollution [105, 106], both are known to alter epigenetics. These discrepancies, while enriching our understanding of epigenetic variability between ethnic groups, also serve as a clarion call for tailored healthcare and research approaches across populations.

In summary, this paper comparatively examined the transferability and health implications of epigenetic GA and GAA in white European and South Asian birth cohorts living in the same country. Two key findings are the consistent associations of parity, GDM, and newborn sex with epigenetic GA in both South Asian and white European cohorts, but with weaker effects observed in the South Asian cohort; and the positive association between epigenetic GA and newborn ponderal index that is exclusive to South Asian newborns. The epigenetic GA clock presents a promising avenue to bridge our understanding of maternal exposures and offspring health outcomes, and these differentiating characteristics of associations across the cohorts, enrich this narrative. Further research in the South Asian population, both adult and pediatric, is needed to strengthen these findings.

### Supplementary Information

Additional file1 Table S1. Quality controls for the inclusion/exclusion of samples and methylation probes. Table S2. Characteristics of CHILD and START epigenetic subsample vs. the overall cohort. Table S3. CpGs associated with gestational age in CHILD or START with FDR adjusted p-value < 0.05. Table S4. Measures of type I error inflation for EWAS association p-values in CHILD and START. Table S5. Significantly associated DMR identified in CHILD. Table S6. Significantly associated DMR identified in START. Table S7. Significantly enriched biological pathways for DMRs associated with chronological GA at birth in CHILD and START. Table S8. GWAS genes associated with T2D or GDM mapped to CpGs significantly associated with GA in START or CHILD. Table S9. Enrichment of GWAS genes associated with T2D or GDM using a two-sample binomial test. Table S10. Differences in gestational age, epigenetic gestational age and acceleration between studies or sex. Table S11. Associations between gestational age, epigenetic gestational age, acceleration of gestational age, and maternal and offspring characteristics in CHILD. Table S12. Associations between gestational age, epigenetic gestational age, acceleration of gestational age, and maternal and offspring characteristics in START

Additional file2 Figure S1. Heatmap of maternal and offspring characteristics in CHILD and START. Heatmap in Panels A-B) display the correlation matrix between the maternal and offspring variables in CHILD and START, respectively. Each cell represents the correlation coefficient between the variables on the corresponding x and y axes, ranging from -1 (strong negative correlation, shown in blue) to +1 (strong positive correlation, shown in red). Cells with a correlation close to 0 are colored in neutral (white), indicating no correlation. The color intensity increases with the strength of the relationship

Additional file3 Figure S2. A Venn Diagram for significant CpGs identified in CHILD, START and Bohlin

Additional file4 Figure S3. Relationship between CpG association effect size and heterogeneity of effect. Panel A) shows the scatterplot of estimated association effect in CHILD (x-axis) and START (y-axis) for CpGs that were significant in the meta-analysis (FDR adjusted p < 0.05) that also were heterogenous in their effects (Heterogeneous p < 0.01) (#CpG = 108); for the same set of CpGs, Panel B) shows the relationship between the absolute difference in beta-values (taken as the mean difference of CHILD and START) and the -log10 Wilcox test p-values, where Wilcox test p-value > 0.05 corresponded to no evidence for difference in distribution of CpG between CHILD and START, and those between 0.05 and 1×10^–5^, 1×10^–5^ and 1×10^–10^, and < 1×10^–10^ as small, medium and large difference. Each CpG that had been mapped to a gene using the “sesame” annotation package was labeled with the corresponding gene. The solid gray line is the best fitted line for the linear relationship between the effect sizes and the dashed gray line represents the reference of y=x

Additional file5 Figure S4. Distributions and scatterplots of CpGs association effect sizes identified in CHILD, START, and Bohlin. Panel A) shows the distribution of estimated association effects for CpGs that were significant in either CHILD or START (#CpG = 3164); Panel B) is the scatterplot of estimated effects in CHILD (x-axis) vs. START (y-axis) for all CpGs present in CHILD, START, and Bohlin (# CpGs = 11337); Panels C) and D) show the scatterplot of estimated effects in CHILD (Panel C) or START (Panel D) with respect to those identified in Bohlin. The solid gray line is the best fitted line for the linear relationship between the effect sizes and the dashed gray line represents the reference of y=x

Additional file6 Figure S5. Distributions of the number of CpGs and DMRs. Panel A) shows the distributions of the number of CpGs that were in the significant DMRs identified in CHILD and START; Panel B) is a scatterplot of the number of DMRs identified in CHILD (x-axis) vs. START (y-axis) across the chromosomes. The dashed line represents the reference of y=x

Additional file7 Figure S6. Overlap of Significant CpG-Mapped Genes with Known Type 2 Diabetes and Gestational Diabetes Genes Identified in GWAS Studies. This karyogram illustrates the chromosomal distribution of previously identified T2D or GDM genes from GWAS catalog in the human genome. Each chromosome is represented by a distinct bar, with the p-arm on the left and the q-arm on the right. Highlighted bands on each chromosome indicate the cytogenetic bands that have been stained and the centromeres are highlighted in red. Genes of interest are marked with black vertical lines along the chromosomes, with only the names of genes that were mapped to CpGs identified in either START (red) or CHILD (green) or both (black) annotated to their respective locations

Additional file8 Figure S7. Scatterplots of maternal age and DNA methylation predicted gestational age. Panel A) shows the relationship stratified by parity with p-value for each level displayed in the left margin, Panel B) shows the overall relationship and the estimated linear model

## Data Availability

The EWAS summary statistics are available from the EWAS catalog at https://www.ewascatalog.org/?study=27717397_gestational_age_ultrasoundestimated_gestational_age. Summary statistics generated in the current study, including a total of 2 primary association studies and the meta-analyzed results are available upon request. All scripts to reproduce the results and figures can be found at https://github.com/WeiAkaneDeng/EpigeneticResearch/tree/main/EpigenticGA.

## Abbreviations

BMI: Body mass index
CpGs: 5'—C—phosphate—G—3'
DNAm: DNA methylation
EWAS: Epigenome-wide association study
GA: Gestational age
GAA: Gestational age acceleration
GDM: Gestational diabetes mellitus
GWAS: Genome-wide association study
OGTT: Oral glucose tolerance test
PI: Ponderal index
T2D: Type 2 diabetes
WHR: Waist-to-hip ratio
