# Supplementary figures and images for: Consistent cord blood DNA methylation signatures of gestational age between South Asian and white European cohorts
Source: Clin Epigenetics. 2024 Jun 6;16:74. doi: 10.1186/s13148-024-01684-0 (PMC11155053; doi:10.1186/s13148-024-01684-0)

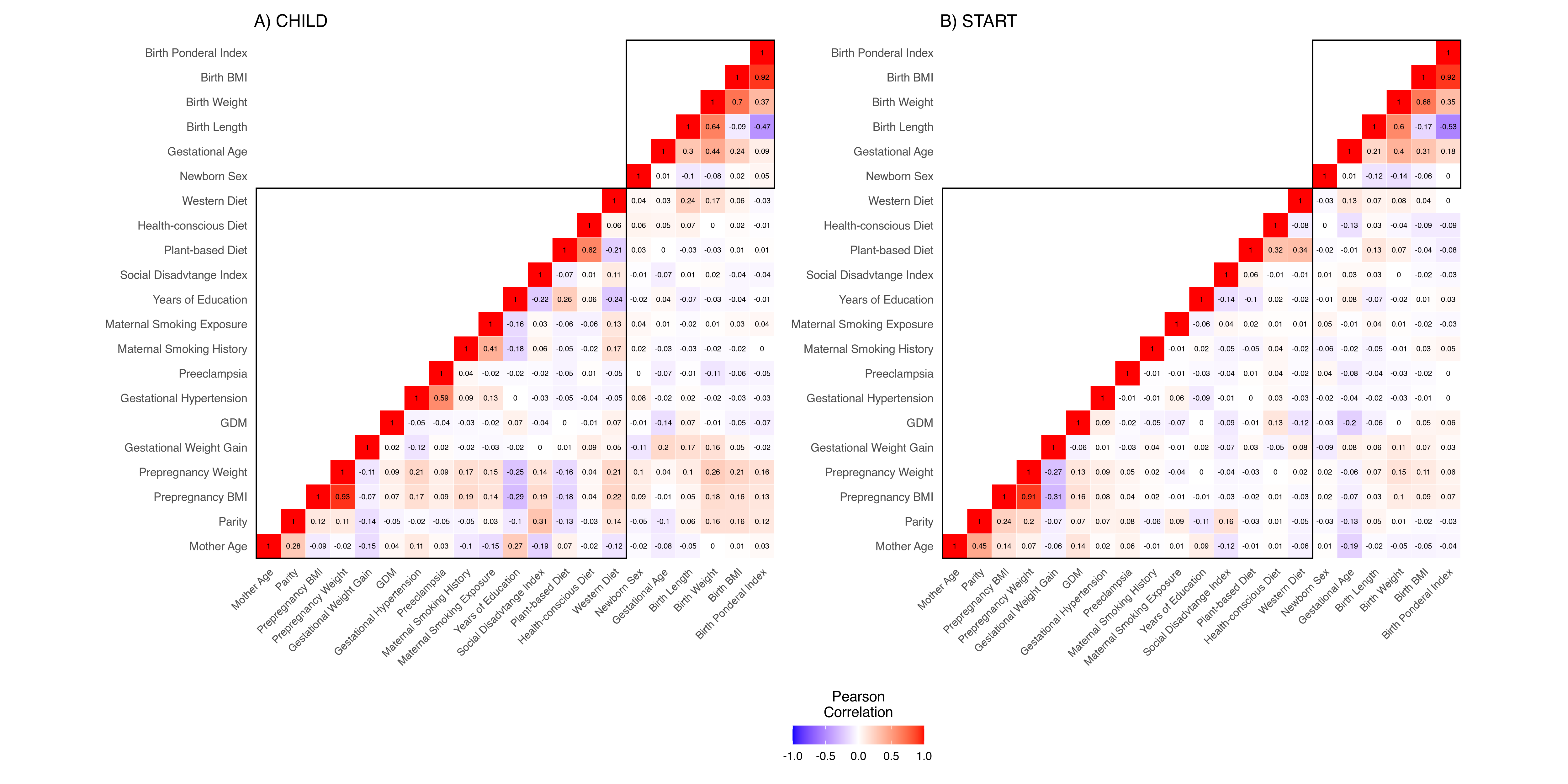

Supplement: Supplementary file 2 — Additional file2 Figure S1. Heatmap of maternal and offspring characteristics in CHILD and START. Heatmap in Panels A-B) display the correlation matrix between the maternal and offspring variables in CHILD and START, respectively. Each cell represents the correlation coefficient between the variables on the corresponding x and y axes, ranging from -1 (strong negative correlation, shown in blue) to +1 (strong positive correlation, shown in red). Cells with a correlation close to 0 are colored in neutral (white), indicating no correlation. The color intensity increases with the strength of the relationship [file 13148_2024_1684_MOESM2_ESM.tiff]

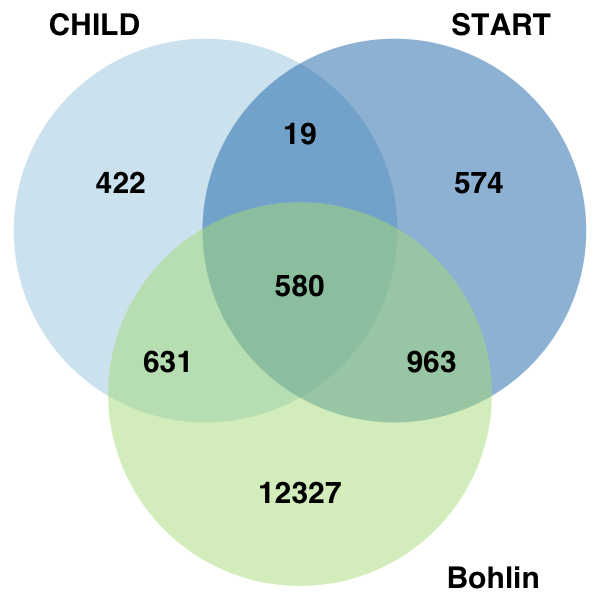

Supplement: Supplementary file 3 — Additional file3 Figure S2. A Venn Diagram for significant CpGs identified in CHILD, START and Bohlin [file 13148_2024_1684_MOESM3_ESM.tiff]

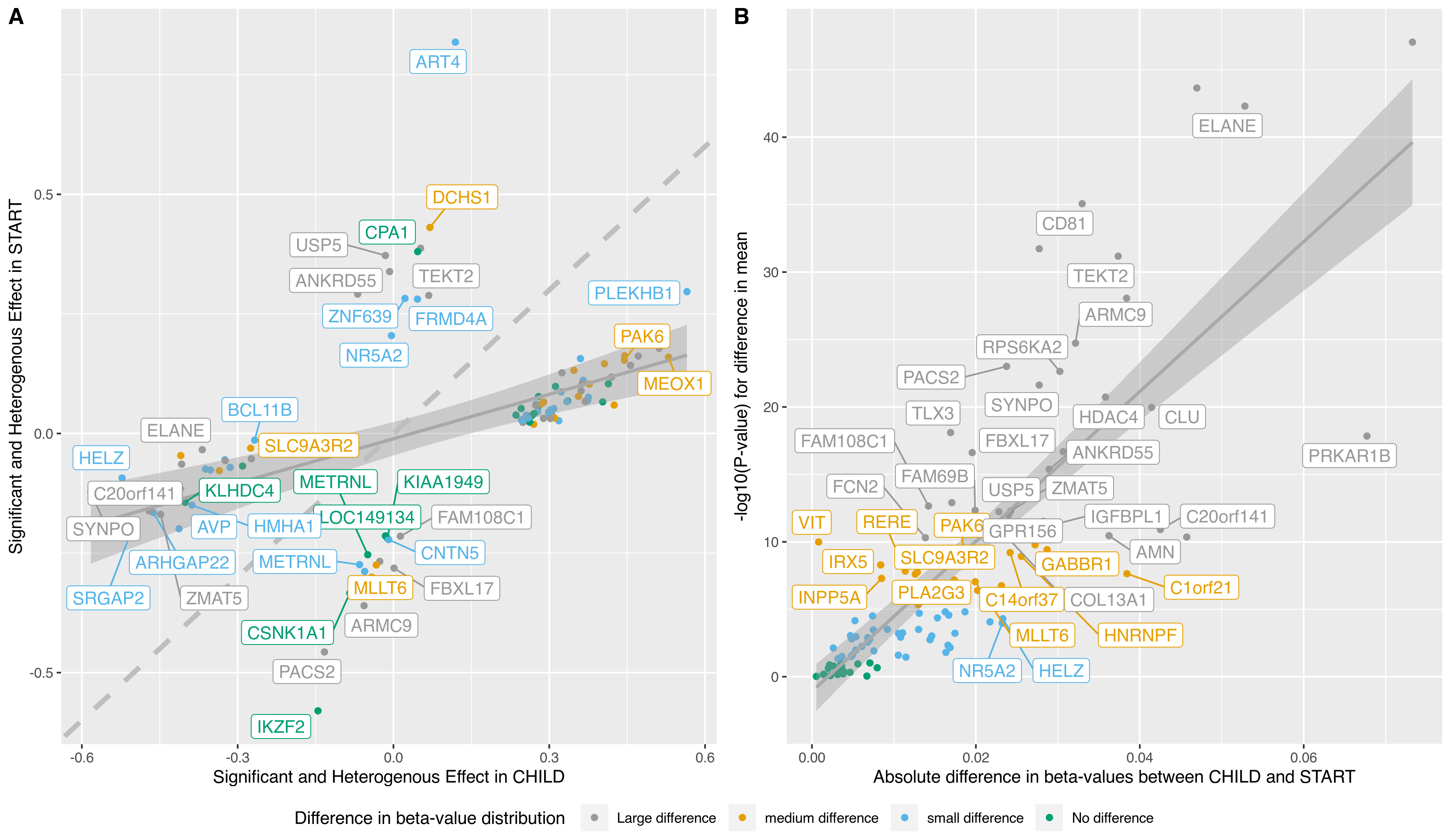

Supplement: Supplementary file 4 — Additional file4 Figure S3. Relationship between CpG association effect size and heterogeneity of effect. Panel A) shows the scatterplot of estimated association effect in CHILD (x-axis) and START (y-axis) for CpGs that were significant in the meta-analysis (FDR adjusted p < 0.05) that also were heterogenous in their effects (Heterogeneous p < 0.01) (#CpG = 108); for the same set of CpGs, Panel B) shows the relationship between the absolute difference in beta-values (taken as the mean difference of CHILD and START) and the -log10 Wilcox test p-values, where Wilcox test p-value > 0.05 corresponded to no evidence for difference in distribution of CpG between CHILD and START, and those between 0.05 and 1×10^–5^, 1×10^–5^ and 1×10^–10^, and < 1×10^–10^ as small, medium and large difference. Each CpG that had been mapped to a gene using the “sesame” annotation package was labeled with the corresponding gene. The solid gray line is the best fitted line for the linear relationship between the effect sizes and the dashed gray line represents the reference of y=x [file 13148_2024_1684_MOESM4_ESM.tiff]

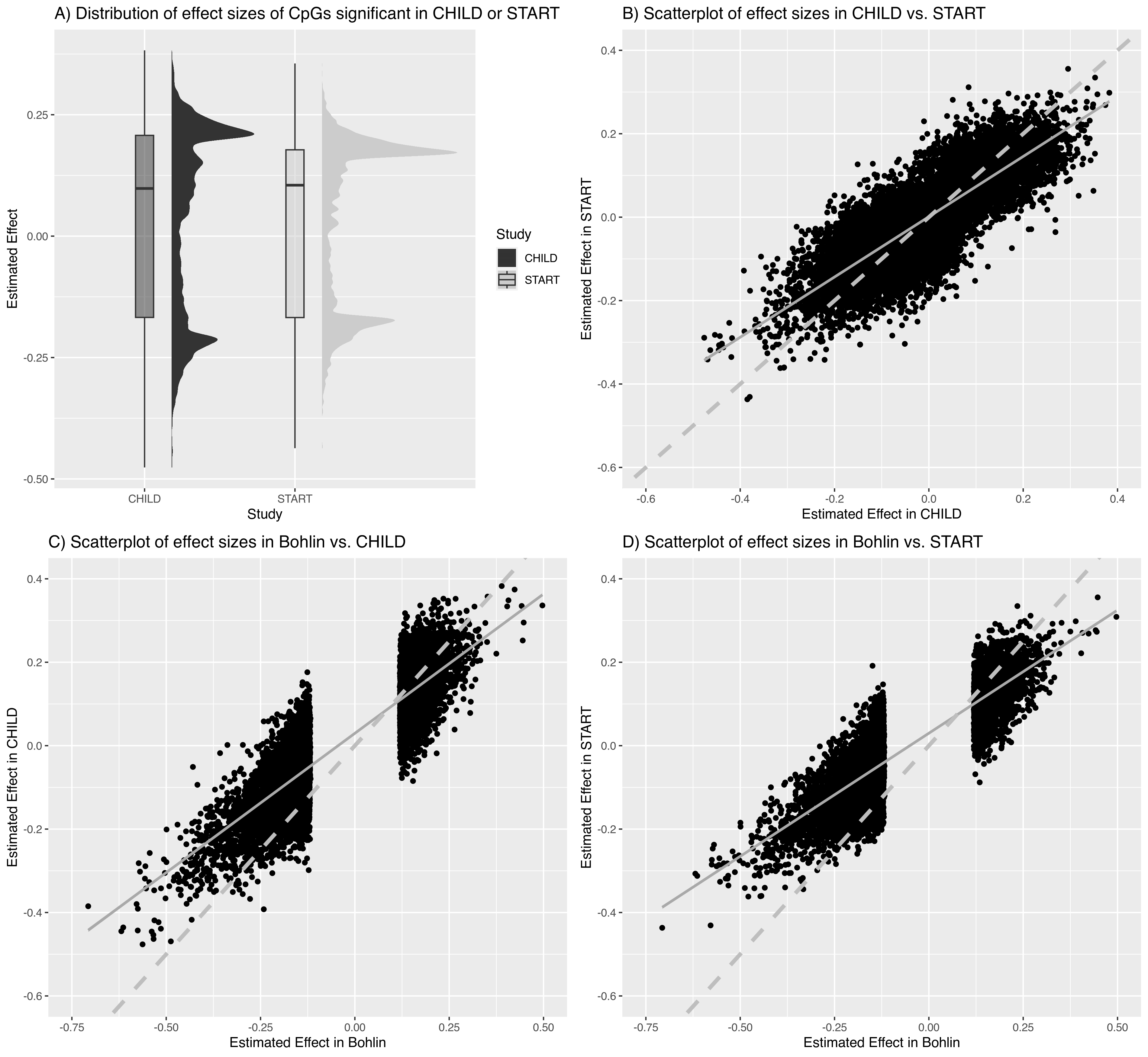

Supplement: Supplementary file 5 — Additional file5 Figure S4. Distributions and scatterplots of CpGs association effect sizes identified in CHILD, START, and Bohlin. Panel A) shows the distribution of estimated association effects for CpGs that were significant in either CHILD or START (#CpG = 3164); Panel B) is the scatterplot of estimated effects in CHILD (x-axis) vs. START (y-axis) for all CpGs present in CHILD, START, and Bohlin (# CpGs = 11337); Panels C) and D) show the scatterplot of estimated effects in CHILD (Panel C) or START (Panel D) with respect to those identified in Bohlin. The solid gray line is the best fitted line for the linear relationship between the effect sizes and the dashed gray line represents the reference of y=x [file 13148_2024_1684_MOESM5_ESM.tiff]

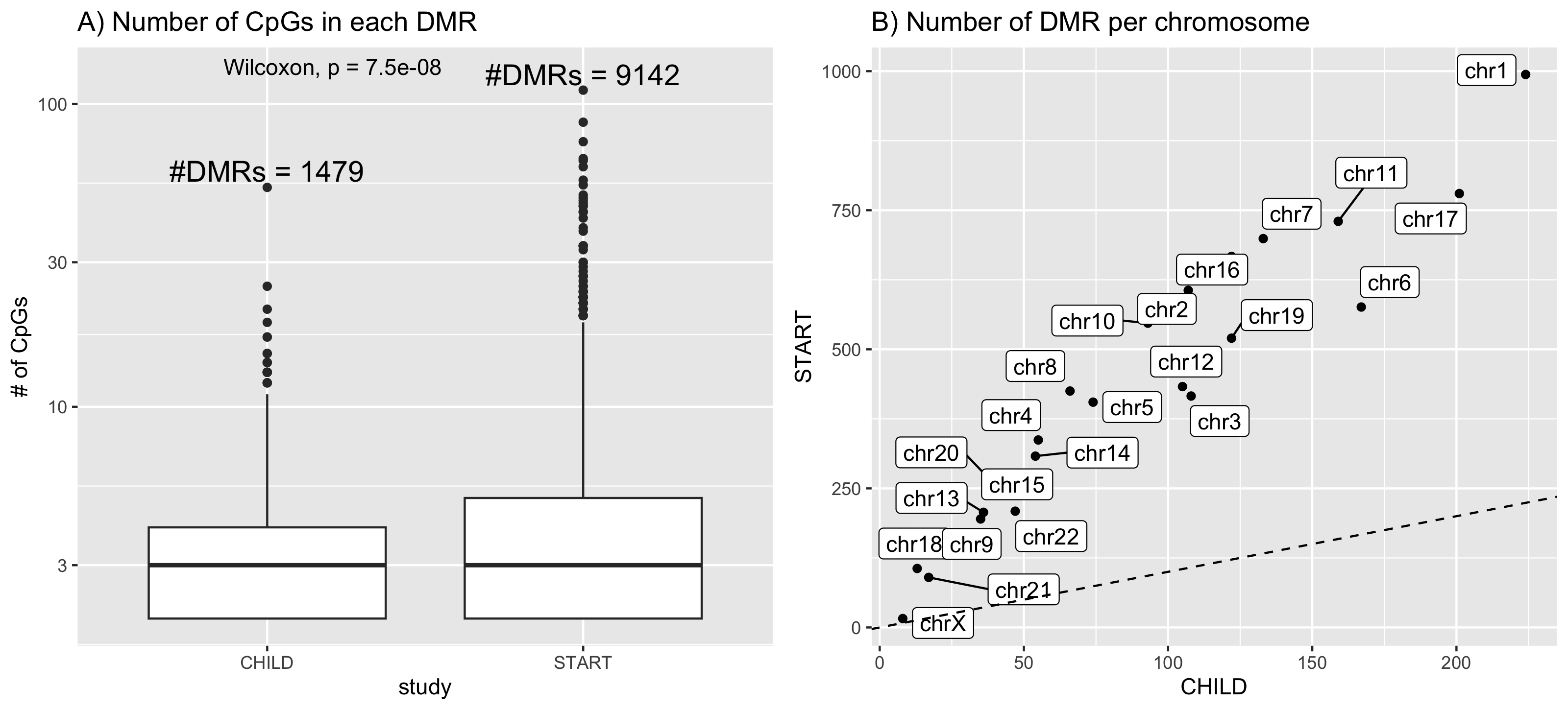

Supplement: Supplementary file 6 — Additional file6 Figure S5. Distributions of the number of CpGs and DMRs. Panel A) shows the distributions of the number of CpGs that were in the significant DMRs identified in CHILD and START; Panel B) is a scatterplot of the number of DMRs identified in CHILD (x-axis) vs. START (y-axis) across the chromosomes. The dashed line represents the reference of y=x [file 13148_2024_1684_MOESM6_ESM.tiff]

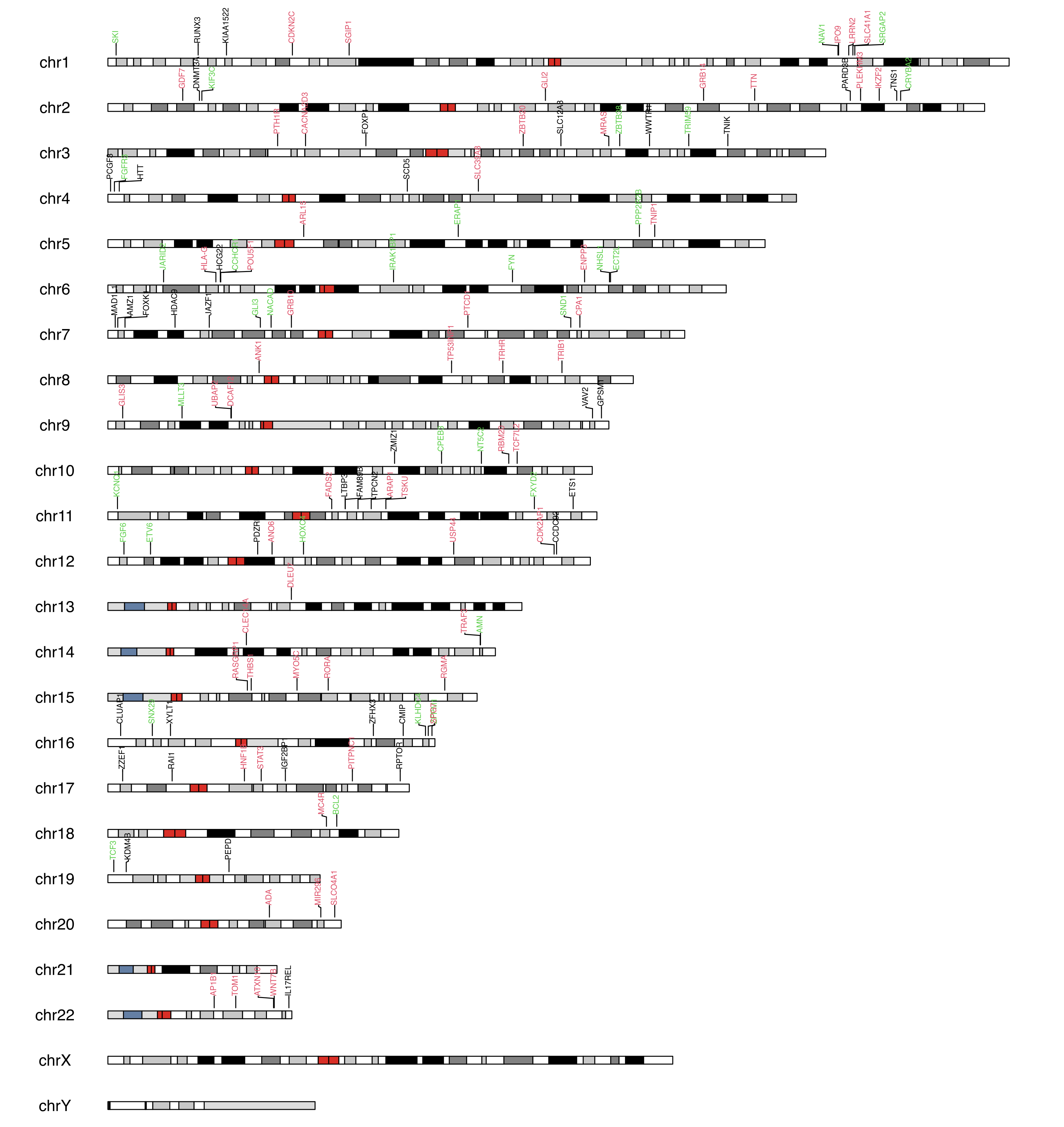

Supplement: Supplementary file 7 — Additional file7 Figure S6. Overlap of Significant CpG-Mapped Genes with Known Type 2 Diabetes and Gestational Diabetes Genes Identified in GWAS Studies. This karyogram illustrates the chromosomal distribution of previously identified T2D or GDM genes from GWAS catalog in the human genome. Each chromosome is represented by a distinct bar, with the p-arm on the left and the q-arm on the right. Highlighted bands on each chromosome indicate the cytogenetic bands that have been stained and the centromeres are highlighted in red. Genes of interest are marked with black vertical lines along the chromosomes, with only the names of genes that were mapped to CpGs identified in either START (red) or CHILD (green) or both (black) annotated to their respective locations [file 13148_2024_1684_MOESM7_ESM.tiff]

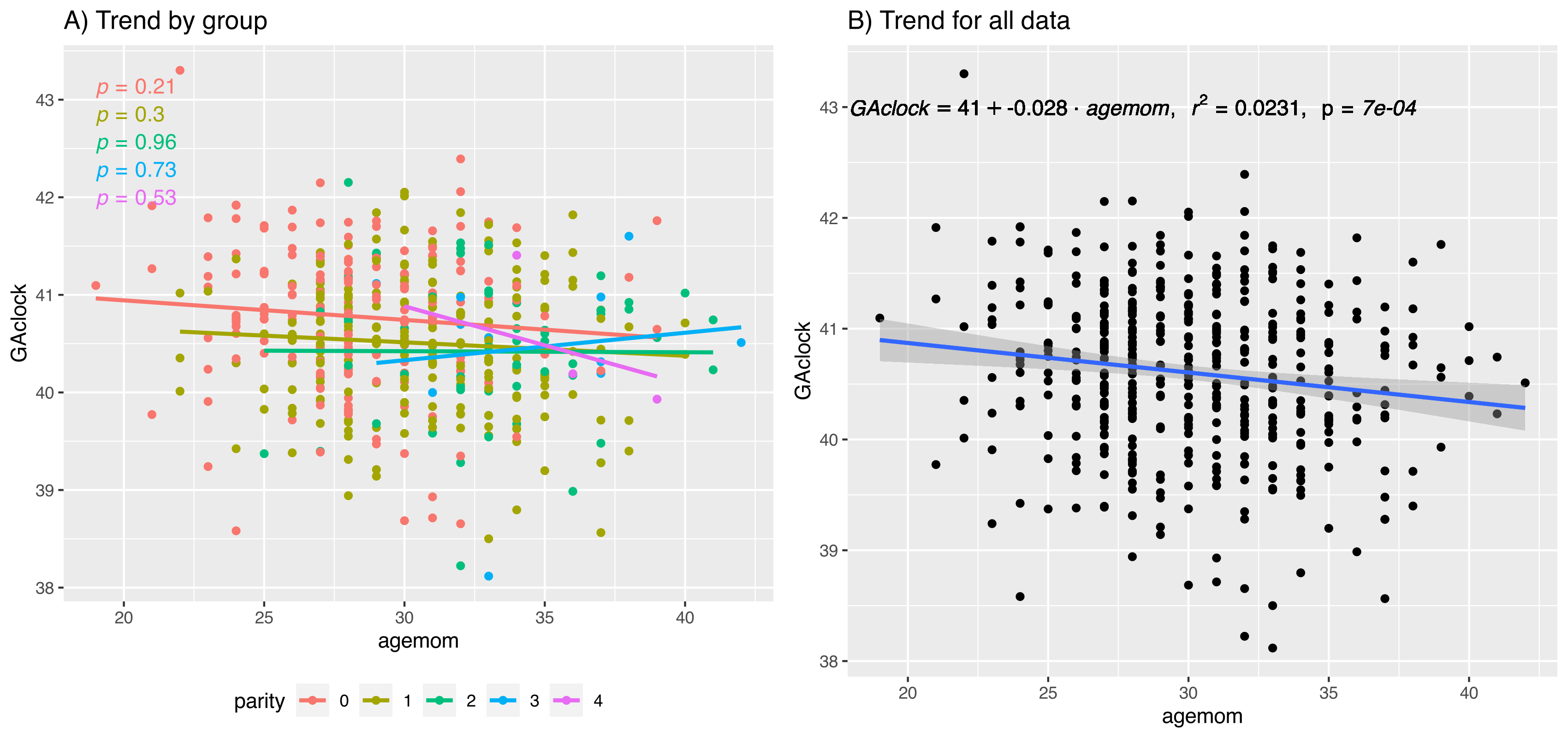

Supplement: Supplementary file 8 — Additional file8 Figure S7. Scatterplots of maternal age and DNA methylation predicted gestational age. Panel A) shows the relationship stratified by parity with p-value for each level displayed in the left margin, Panel B) shows the overall relationship and the estimated linear model [file 13148_2024_1684_MOESM8_ESM.tiff]
